# Gong’s acupuncture treatment for mid pregnancy insomnia and anxiety: A case report

**DOI:** 10.1097/MD.0000000000040698

**Published:** 2024-11-22

**Authors:** Kuilin Wu, Ailan Yu, Zhong Liu, Xiujuan Gao, Yonghong Ding, Yufang Shi, Zongwang Zhang

**Affiliations:** a Shandong First Medical University, Jinan, Shandong, China; b Liaocheng People’s Hospital, Liaocheng, Shandong, China.

**Keywords:** anxiety, case report, Gong’s brain acupuncture, insomnia, pregnancy

## Abstract

**Rationale:**

Gong’s brain acupuncture (GBA) is a acupuncture technique that restores the balance of the central nervous system by stimulating specific acupoints on the skull to transmit stimulation to the nerves. Insomnia during pregnancy is an increasingly concerning issue, and GBA provides new solutions.

**Patient’s concerns:**

The patient, a 26-year old woman at 26 + 1 weeks of pregnancy, presented with unexplained insomnia for 3 weeks. Her symptoms included difficulty initiating sleep, light sleep, and intermittent wakefulness throughout the night.

**Diagnoses:**

The patient was diagnosed with primary insomnia, scoring 16 on the Pittsburgh sleep quality index, which indicates poor sleep quality. Her self-rating anxiety scale (SAS) score was 49 points, suggesting that she had not yet reached a state of mild anxiety.

**Interventions:**

The patient received 15 sessions of GBA treatment.

**Outcomes:**

One month after treatment completion, the patient’s Pittsburgh sleep quality index score improved to 3 points, indicating good sleep quality. Her self-rating anxiety scale score decreased to 39 points.

**Lessons:**

This case report demonstrated the successful treatment of mid pregnancy insomnia with GBA, providing preliminary evidence for further research on the therapeutic potential of GBA. However, further research is still needed to enhance persuasiveness.

## 1. Introduction

Insomnia during pregnancy is a common issue encountered by expectant mothers. Statistics indicate that approximately 49% of pregnant women experience sleep disturbances during the second trimester, which may include difficulties in falling asleep, frequent nocturnal awakenings, and early morning risings.^[1]^ Pregnancy-related insomnia not only impairs the physical well-being of pregnant women but may also result in adverse pregnancy outcomes.^[2,3]^ Given the unique nature of pregnancy, pharmacological treatment for insomnia in pregnant women must be approached with caution, as numerous medications may pose potential risks to the developing fetus. Non-pharmacological interventions should be attempted before considering drug treatment, such as improving sleep hygiene, relaxation techniques, cognitive behavioral therapy, and acupuncture and moxibustion.^[4,5]^ If these approaches prove ineffective, healthcare providers may consider prescribing medications under close supervision, including antihistamines, benzodiazepines, non-benzodiazepines, antidepressants, and melatonin.^[5,6]^

As a crucial component of traditional Chinese medicine, acupuncture therapy has demonstrated remarkable efficacy in treating a wide range of diseases. Previous studies have indicated that acupuncture is highly effective in treating insomnia among pregnant women, effectively alleviating insomnia symptoms, improving pregnancy outcomes, and exhibiting a high level of safety.^[7–9]^ The mechanism behind this may involve significantly enhancing the quality of sleep among pregnant women by increasing melatonin secretion.^[10]^ Furthermore, acupuncture therapy can also improve mental health during pregnancy.^[11,12]^ For instance, auricular acupuncture has been found to significantly reduce prenatal anxiety levels.^[13]^

In China, Gong’s brain acupuncture (GBA) represents a novel treatment modality that has exhibited considerable therapeutic efficacy in clinical settings. GBA has been proven effective in treating various conditions, including insomnia,^[14]^ headaches,^[15]^ sequelae of stroke,^[16]^ pain,^[17]^ and intractable hiccups.^[18]^ Recently, we successfully treated a patient in the second trimester of pregnancy, improving her sleep quality through GBA therapy. We hope that this study will provide preliminary evidence to support the use of GBA in treating insomnia and anxiety during pregnancy.

## 2. Case report

On March 16, 2024, Liaocheng People’s Hospital admitted a pregnant woman with insomnia to our hospital. This 26-year old housewife was 26 weeks and 1 day pregnant, and this was her second child. She had experienced insomnia for the past 3 weeks. Her condition was primarily characterized by difficulty falling asleep, a lack of restful sleep, and occasional bouts of insomnia that lasted throughout the night. The patient recounted that she had barely slept for 3 consecutive nights, a period that left her severely mentally and physically drained, ultimately compelling her to seek medical assistance. She disclosed that due to a family member’s hospitalization for treatment last year, she had endured insomnia for approximately 3 months and had resorted to traditional Chinese medicine to alleviate her symptoms. Expressing her concern over the potential duration of her current insomnia and its potential impact on her unborn child, she confessed her hesitation to take medication. Furthermore, there was no known family history of hereditary disease.

To quantify her sleep condition, we administered the Pittsburgh sleep quality index (PSQI), which yielded a score of 16, indicating poor sleep quality, sleep disorders, and the need for intervention to avoid secondary health problems and daytime functional impairment.^[19]^ self-rating anxiety scale (SAS) was used to assess her anxiety level, and a score of 49 suggested that her anxiety was below the threshold for a clinical diagnosis, indicating either mild anxiety symptoms or no significant anxiety symptoms.^[20]^

**Figure 1.**
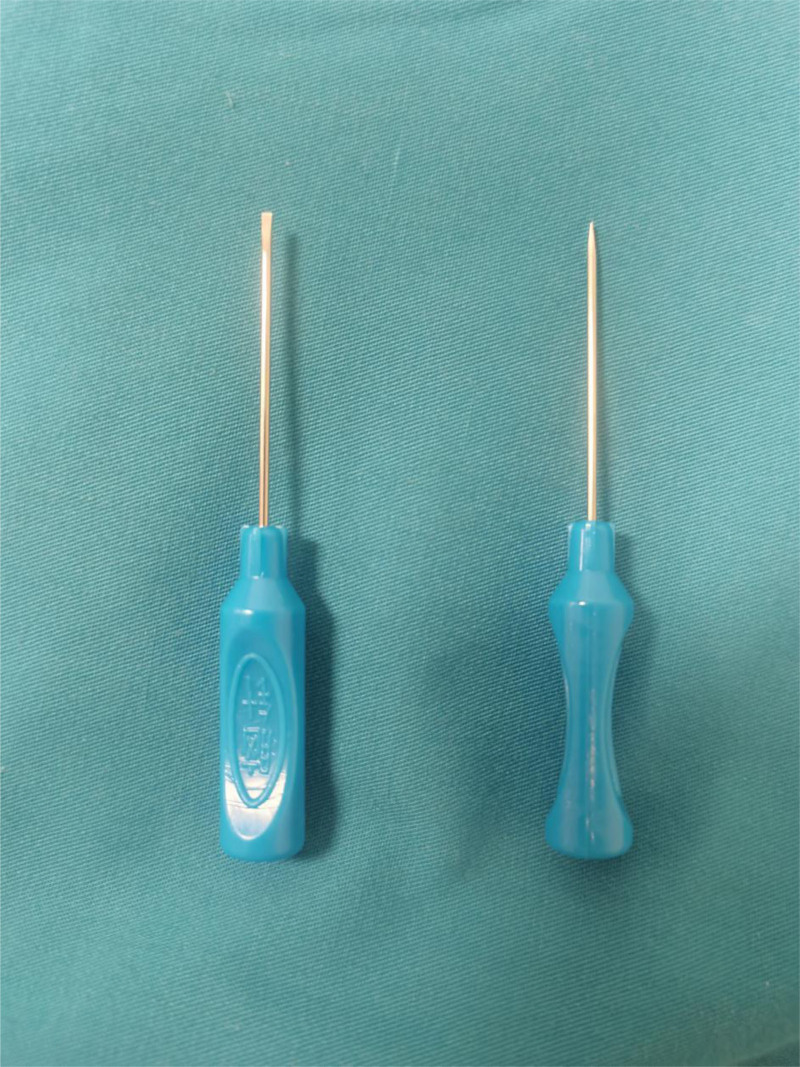
Gong’s brain needle.

GBA, invented by Professor Gong Changxiang (Fig. 1), is a technique that involves acupuncture treatment of the occipital protuberance area to regulate and balance the descending facilitation and inhibition of the nervous system, thereby improving various functional disorders.^[18]^ The course of treatment included 15 sessions, with the first 5 sessions lasting for 5 consecutive days and the remaining 10 sessions performed every other day. After completing the treatment course, the patient reported improvement in her insomnia. We conducted a telephone follow-up 1 month after the end of treatment and reassessed the patient using the PSQI and SAS. Her PSQI score decreased to 3 points, and her SAS score dropped to 39 points, indicating a complete recovery from her sleep disorder and a significant improvement in her mental state.

## 3. Data collection tools

PSQI is a method used to evaluate sleep quality over the past month. In this study, we primarily utilized 18 items that contribute to the scoring. The items mentioned above are categorized into 7 dimensions based on their types, including sleep quality, sleep latency, sleep duration, sleep efficiency, sleep disturbances, use of hypnotic medication, and daytime dysfunction. The score ranges from 0 to 21, with higher scores indicating more severe insomnia. It takes participants 5 to 10 minutes to complete the questionnaire. A score above 7 suggests poor sleep quality, while a score below 7 indicates good sleep quality.^[21]^ SAS is a self-rating scale with a total of 20 items, using a 4-point rating scale to primarily assess the frequency of defined symptoms. A standard score of <50 indicates no anxiety, while a score of ≥50 suggests possible anxiety, with higher scores indicating more severe anxiety.^[22]^

## 4. Treatment plan

**Figure 2.**
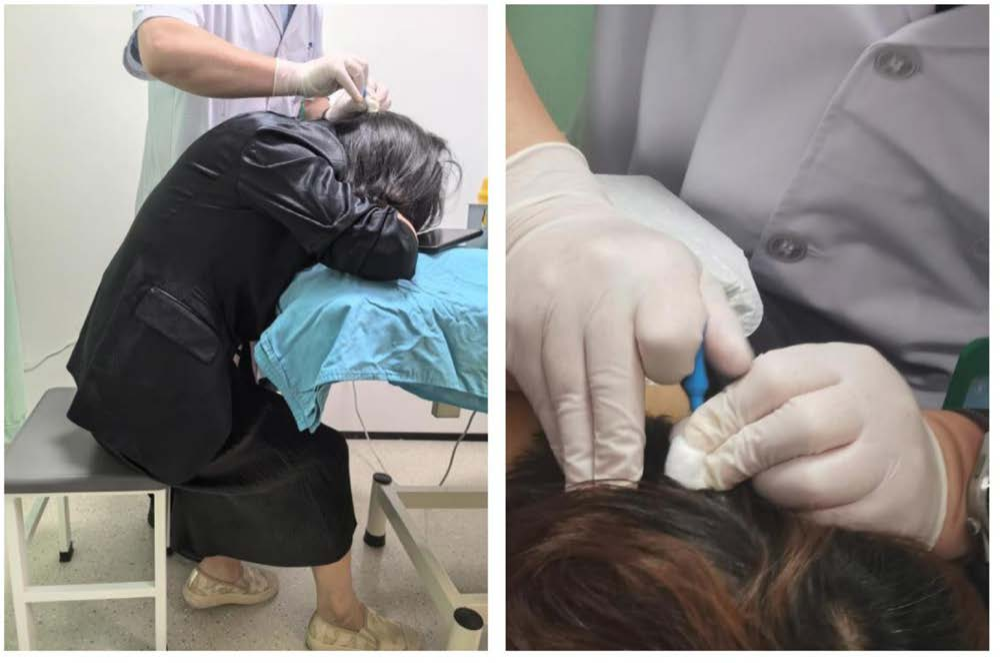
Treatment operation diagram.

Seated comfortably, the patient laid her crossed hands on the treatment table, allowing her forehead to rest lightly on the backs of their hands. The physician began by identifying a point located 0.5 cm above the occipital protuberance at the midline of the posterior aspect of the skull. Subsequent acupoints were positioned 2 cm apart from one another. Following standard disinfection procedures and donning gloves, lidocaine administered 0.3 mL of administered for local anesthesia. Next, the GBA was swiftly inserted vertically toward the bone surface. The needle was manipulated back and forth while being pressed downward until its tip penetrated the bone by approximately 1 mm, at which point it was anchored securely. Thereafter, the GBA was carefully removed (Fig. 2), and the treatment point was compressed with a cotton ball to prevent bleeding. The treatment session was deemed complete once there was no sign of bleeding at the needle insertion site after 5 minutes. Typically, each treatment session lasted for approximately 60 seconds.^[18]^

## 5. Patient’s perspective

The patient felt that our treatment was very effective and presented us with a banner to express gratitude.

## 6. Discussion

Mid pregnancy insomnia is a common problem with diverse possible causes. Hormonal changes are a major cause of insomnia. During mid pregnancy, significant changes in hormone levels in the bodies of pregnant women may affect their sleep patterns. Second, as pregnancy progresses, the pregnant woman’s belly gradually increases, which may lead to discomfort in the waist and legs, thereby affecting the quality of sleep. Frequent urination is a common cause of insomnia, especially at night. Frequent waking can disrupt the sleep cycle, making it difficult for pregnant women to obtain sufficient sleep. In addition, changes in hormone levels may lead to more dreams in pregnant women, which can also affect their sleep quality. Finally, many pregnant women may feel anxious about their upcoming babies and parenting responsibilities, which can also lead to insomnia.^[1,2,23]^ This mother reported experiencing insomnia without any underlying cause, and we speculate that it may be due to changes in pregnancy hormones that cause insomnia. Previous studies have shown that insomnia in mid pregnancy may not only lead to anxiety but may also be an early signal of depression.^[24–26]^ Therefore, treatment of insomnia during mid pregnancy is particularly important.

In this context, the research and clinical practice of Mr. Gong Changxiang offer a novel viewpoint. With over 2 decades of dedication to clinical research on pain, Gong synthesized and delved into various theories through extensive clinical experience. He posited that the root causes of functional disorders, such as pain and insomnia, stem from malfunctioning of the nervous system. His approach involves adjusting the functionality of the nervous system by stimulating bone tissue near the external occipital protuberance through acupuncture, thereby fostering disease recovery. This form of brain acupuncture therapy, also known as Gong’s nerve balance theory and technology, was initially conceptualized by Professor Zhou Liqun of the Beijing University of Traditional Chinese Medicine. Professor Zhou believes that Gong’s neural balance theory is grounded in neuroscience and employs traditional Chinese acupuncture techniques. Although the precise mechanism of GBA remains elusive, there are international medical reports documenting its therapeutic benefits in treating refractory hiccups,^[18]^ tension headaches,^[15]^ cancer-related pain,^[17]^ and other conditions.

Recent studies indicate a strong likelihood that the intracranial dural venous sinus resides precisely beneath the occipital protuberance.^[27]^ This finding prompted us to hypothesize that stimulation of the occipital protuberance via GBA is transmitted to the brain through these venous sinuses, triggering a therapeutic mechanism. However, it is imperative to note that further research is required to substantiate this theory with concrete evidence. Another compelling explanation for the efficacy of GBA stems from the hypothesis put forth by renowned Chinese neurophysiologist Zhang Xiangtong in the mid-1960s. He postulated that “acupuncture analgesia is achieved through the central nervous system’s intricate interplay, processing, and integration of signals originating from the pain source at acupuncture points.” This theory has subsequently been validated by numerous experimental studies,^[28]^ lending credence to the belief that GBA activates the inhibition of arousal structures by needle stimulation of bone tissue. However, the precise mechanism underlying this phenomenon remains a subject of ongoing research.

In general, the treatment of insomnia in the second trimester of pregnancy requires a comprehensive consideration of various factors. GBA therapy does not require needle retention after acupuncture, and the treatment time is relatively short, which can reduce the fear and discomfort of pregnant women during treatment and increase compliance. Compared with CBT-I, GBA therapy is simpler and less expensive, and has the significance of being promoted.

## 7. Conclusion

In summary, this report presents preliminary clinical evidence for GBA in the treatment of insomnia, demonstrating its potential as an effective treatment method. However, to comprehensively verify its efficacy, standardized, rigorous, and higher-level evidence, randomized controlled trials are still needed.

## Author contributions

**Data curation:** Kuilin Wu, Ailan Yu, Zhong Liu, Xiujuan Gao.

**Formal analysis:** Kuilin Wu.

**Funding acquisition:** Zongwang Zhang.

**Investigation:** Yufang Shi.

**Project administration:** Yonghong Ding.

**Resources:** Xiujuan Gao, Zongwang Zhang.

**Supervision:** Ailan Yu, Zhong Liu, Yufang Shi, Zongwang Zhang.

**Writing – original draft:** Kuilin Wu.

**Writing – review & editing:** Ailan Yu, Zhong Liu, Xiujuan Gao, Yonghong Ding, Zongwang Zhang.
